# Caspase-12 and the Inflammatory Response to *Yersinia pestis*

**DOI:** 10.1371/journal.pone.0006870

**Published:** 2009-09-01

**Authors:** Bart Ferwerda, Matthew B. B. McCall, Maaike C. de Vries, Joost Hopman, Boubacar Maiga, Amagana Dolo, Ogobara Doumbo, Modibo Daou, Dirk de Jong, Leo A. B. Joosten, Rudi A. Tissingh, Frans A. G. Reubsaet, Robert Sauerwein, Jos W. M. van der Meer, André J. A. M. van der Ven, Mihai G. Netea

**Affiliations:** 1 Department of Internal Medicine, Radboud University Nijmegen Medical Center, Nijmegen, The Netherlands; 2 Nijmegen Institute for Infectious Inflammation & Immunity (N4i), Radboud University Nijmegen Medical Center, Nijmegen, The Netherlands; 3 Department of Medical Microbiology, Radboud University, Nijmegen Medical Centre, Nijmegen, The Netherlands; 4 Laboratory for Infectious Diseases and Perinatal Screening, Center for Infectious Disease Control Netherlands, National Institute for Public Health and the Environment (RIVM), Bilthoven, The Netherlands; 5 Malaria Research & Training Centre, Faculty of Medicine, University of Bamako, Bamako, Mali; 6 Department of Gastroenterology and Hepatology, Radboud University Nijmegen Medical Center, Nijmegen, The Netherlands; University of California Merced, United States of America

## Abstract

**Background:**

Caspase-12 functions as an antiinflammatory enzyme inhibiting caspase-1 and the NOD2/RIP2 pathways. Due to increased susceptibility to sepsis in individuals with functional caspase-12, an early-stop mutation leading to the loss of caspase-12 has replaced the ancient genotype in Eurasia and a significant proportion of individuals from African populations. In African-Americans, it has been shown that caspase-12 inhibits the pro-inflammatory cytokine production.

**Methodology/Principal Findings:**

We assessed whether similar mechanisms are present in African individuals, and whether evolutionary pressures due to plague may have led to the present caspase-12 genotype population frequencies. No difference in cytokine induction through the caspase-1 and/or NOD2/RIP2 pathways was observed in two independent African populations, among individuals with either an intact or absent caspase-12. In addition, stimulations with *Yersinia pestis* and two other species of *Yersinia* were preformed to investigate whether caspase-12 modulates the inflammatory reaction induced by *Yersinia*. We found that caspase-12 did not modulate cytokine production induced by *Yersinia* spp.

**Conclusions:**

Our experiments demonstrate for the first time the involvement of the NOD2/RIP2 pathway for recognition of *Yersinia*. However, caspase-12 does not modulate innate host defense against *Y. pestis* and alternative explanations for the geographical distribution of caspase-12 should be sought.

## Introduction

Caspases are cysteine proteases that are involved in apoptosis and inflammation [1]–[3]. Three human caspases involved in the inflammation pathway are known, namely caspase-1, 4, and 5 [2], [3]. In other mammals such as mice and chimpanzees, another caspase with modulatory effects on inflammation is caspase-12. In humans, a truncated non-functional form of caspase-12 due to a mutation resulting in an early stop codon has largely replaced the ancient genotype [4]. Genetic and population analysis revealed that the functional form of caspase-12 is present in 20 to 30% of the African populations, while completely absent in Europe and Asia [5], [6]. Due to this particular worldwide distribution and its involvement in inflammation, caspase-12 has been proposed as an exquisite example of an immune gene changed under evolutionary pressure.

Functionally, caspase-12 has been reported to be involved both in the apoptotic and inflammatory pathways [7]. Whole blood stimulation with lipopolysaccharide (LPS) showed that individuals bearing the functional caspase-12 were hypo-responsive. LPS, an outer cellular membrane structure found on Gram-negative bacteria is recognized by our innate immune system [8]. The innate immune system recognizes specific pathogen structures, such as LPS through pattern recognition receptors (PRR) like toll-like receptors (TLRs) [9]. TLR4 is the most important PRR involved in the recognition of LPS, TLR2 recognizes bacterial lipopeptides, and the NOD2 receptor recognizes peptidoglycans. PRR engagement induces production of proinflammatory cytokines such as TNF-α and IL-1β. The IL-1β activation requires cleavage by another inflammatory caspase termed IL-1β converting enzyme or caspase-1 [10].

After LPS stimulation, individuals with a functional caspase-12 produced lower TNF-α and IL-1β levels compared to individuals bearing only the truncated form of caspase-12 [11]. It has been therefore proposed that caspase-12 has anti-inflammatory effects by inhibiting both the NFκB and caspase-1 pathways [12]. As a consequence of the altered NFκB and caspase-1 pathway stimulation in individuals bearing functional caspase-12, the clearance of Gram-negative bacteria may be defective and eventually this could result in a life threatening bacterial infection [13], [14]. A small clinical study among African Americans showed that the pressure of a functional caspase-12 was accompanied by an increased susceptibility to sepsis [15]. These findings were supported by a sepsis mouse model showing that caspase-12 mice had a higher bacterial count and lower pro-inflammatory cytokine production during sepsis [11].

Evolutionary studies [6], [16] determined that the loss of functional caspase-12 in Caucasian and Asian populations was caused by recent positive selection. The loss of function of caspase-12 is due to truncation of the protein, rather then modulation of mRNA transcription. These data combined with the correlation of caspase-12 with susceptibility to sepsis, lead to the hypothesis that the loss of functional caspase-12 was due to the increasing pressure of sepsis during the human migration towards Europe and Asia [6]. After blood stimulation of individuals bearing the functional and non-functional caspase-12 with LPS it was shown that the non-functional caspase-12 protein was not transcriped [11]. This indicates that early stop codon results in a loss of function of the caspase-12. However, the assumption that the loss of function of capase-12 involved in the innate immune response are based on a single study in a small number of African Americans, but no data on the function of caspase-12 in African populations are available [16].

This is essential for the understanding of how the selection has driven selection of caspase-12 genotypes during human history. In the present study we assessed the impact of caspase-12 genotypes on inflammation in two African populations, and the potential effect of caspase-12 on a microorganism known to have exerted intense evolutionary pressure on European and Asian populations: *Yersinia pestis*, the agent of bubonic plague.

## Results

### Functional and non-functional caspase-12 frequencies in Mali volunteers

To evaluate the effect of non-functional and functional caspase-12 genotypes on proinflammatory cytokines, blood was initially collected from 50 healthy Mali volunteers. Thirthy-three of the volunteers were homozygous (66%) for the non-functional caspase-12 allele, 16 volunteers were heterozygous (32%), and one (2%) was homozygous for the functional caspase-12 allele (Table 1). These frequencies of the caspase-12 genotypes were in line with previous reports [5].

**Table 1:** Caspase-12 genotypes and allele frequency in two cohorts of Mali volunteers, one collected in 2006 and one in 2007.

	Non-functional	Functional	Functional		Genotype frequency (%)			Allele frequency	
	TGA S/S	(T/C)GA S/L	CGA L/L	Total	T/T	C/T	C/C	T	C
2006 a	33	16	1	50	66.00	32.00	2.00	0.82	0.18
2007 a	24	22	1	47	51.06	46.81	2.13	0.74	0.26
Total a,b	57	38	2	97	58.76	39.18	2.06	0.78	0.22

a Population do not violate Hardy-Weinberg equilibrium.

b Distribution of Caspase-12 genotypes between 2006 and 2007 populations did not differ (χ^2^-test).

### Pro-inflammatory cytokine production of the caspase-12 genotypes

**Figure 1:**
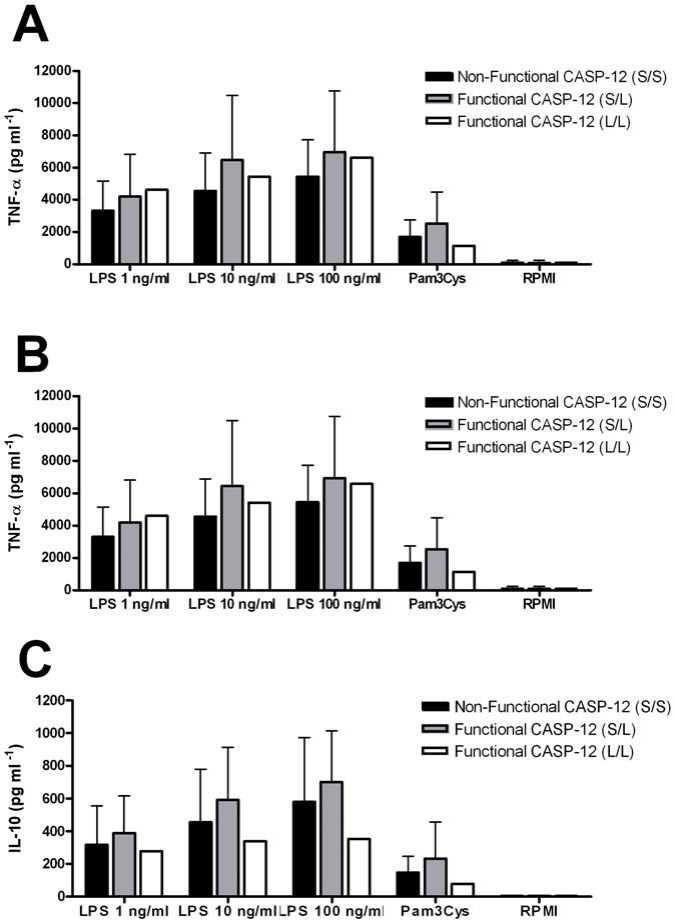
In-vitro cytokine measurements after whole blood LPS and Pam3Cys stimulation between the caspase-12 genotypes from the volunteers enrolled in 2006. Stimulation was performed with 1, 10 and 100 ng/ml LPS (*E. coli*) and 10 µg/ml Pam3Cys. TNF (A), IL-1β (B) and IL-10 (C) were measured by ELISA in the supernatant after 24 hour stimulation. Volunteers were grouped as homozygous bearing the nonfunctional caspase-12 genotype (S/S), heterozygous bearing the nonfunctional and functional caspase-12 (S/L), and homozygous bearing the functional caspase-12 (L/L). Numbers of volunteers included for each cytokine are S/S = 33, S/L = 16 and L/L = 1. Values represent mean + SD for each group of volunteers. P-values for differences between caspase-12 genotype were calculated with the Mann-Whitney test. *P<0.05.

Stimulation of whole blood was performed with either the TLR4 ligand LPS (concentrations 1, 10 and 100 ng/ml) or Pam3Cys (10 µg/ml), a TLR2 specific ligand. Similar levels of TNF-α between caspase-12 genotypes were observed between the groups (Figure 1A). Surprisingly, the IL-1β production was even increased in individuals heterozygous for the functional caspase-12 allele, for all LPS concentrations tested (Mann-Whitney, P<0.05, figure 1B). In addition, the individual homozygous for the functional caspase-12 allele showed no evidence of inhibition of the production of pro-inflammatory cytokines (Figure 1). The release of the anti-inflammatory cytokine IL-10 was independent of the caspase-12 genotype (Figure 1C).

### Replication within second field study of previous findings

**Figure 2:**
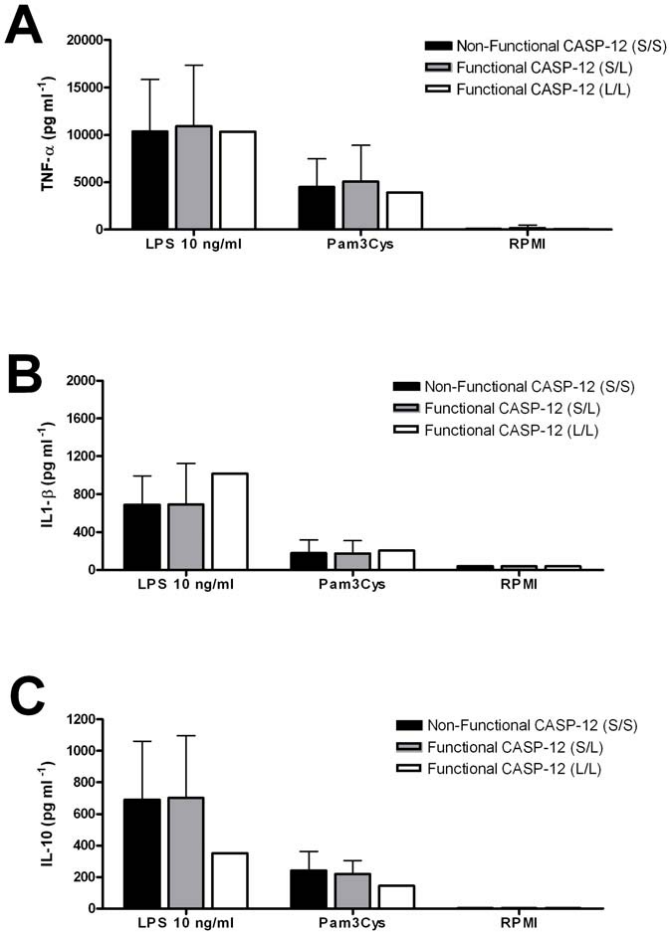
In-vitro cytokine measurements after whole blood LPS and Pam3Cys stimulation between the caspase-12 genotypes from the volunteers enrolled in 2007. Stimulation with LPS was performed with 10 ng/ml. TNF (A), IL-1β (B) and IL-10 (C) were measured by ELISA after 24-hour stimulation. Volunteers were grouped as homozygous bearing the nonfunctional caspase-12 genotype (S/S), heterozygous bearing the nonfunctional and functional caspase-12 (S/L) and homozygous that bear the functional caspase-12 (L/L). Numbers of volunteers included for each cytokine are S/S = 24, S/L = 22 and L/L = 1. Values represent mean + SD for each group of volunteers. P-values for differences between caspase-12 genotype were calculated with the Mann-Whitney test.

Because the result of our initial study clearly contradicted one earlier report of inhibition of the pro-inflammatory response by caspase-12, we initiated a second field study to assess the function of caspase-12 [11]. During this second study we collected blood from 47 Mali volunteers and determined their caspase-12 genotype (volunteers 2007, Table 1). Twenty-four individuals were homozygous (51%) for the non-functional caspase-12 allele, 22 heterozygous (47%), and one volunteer was homozygous (2%) for the functional caspase-12 allele (Table 1). No difference was found between cells isolated from individuals homozygous for the non-functional caspase-12 allele and from the heterozygous volunteers, when production of TNF-α, IL-1β or IL-10 was measured (Figure 2). No differences for the production of IL-1β were found between the groups in this second study. The cytokine production of cells isolated from the volunteer homozygous for the functional caspase-12 was comparable with the other genotypes (Figure 2 B and C).

### Yersinia spp stimulations

**Figure 3:**
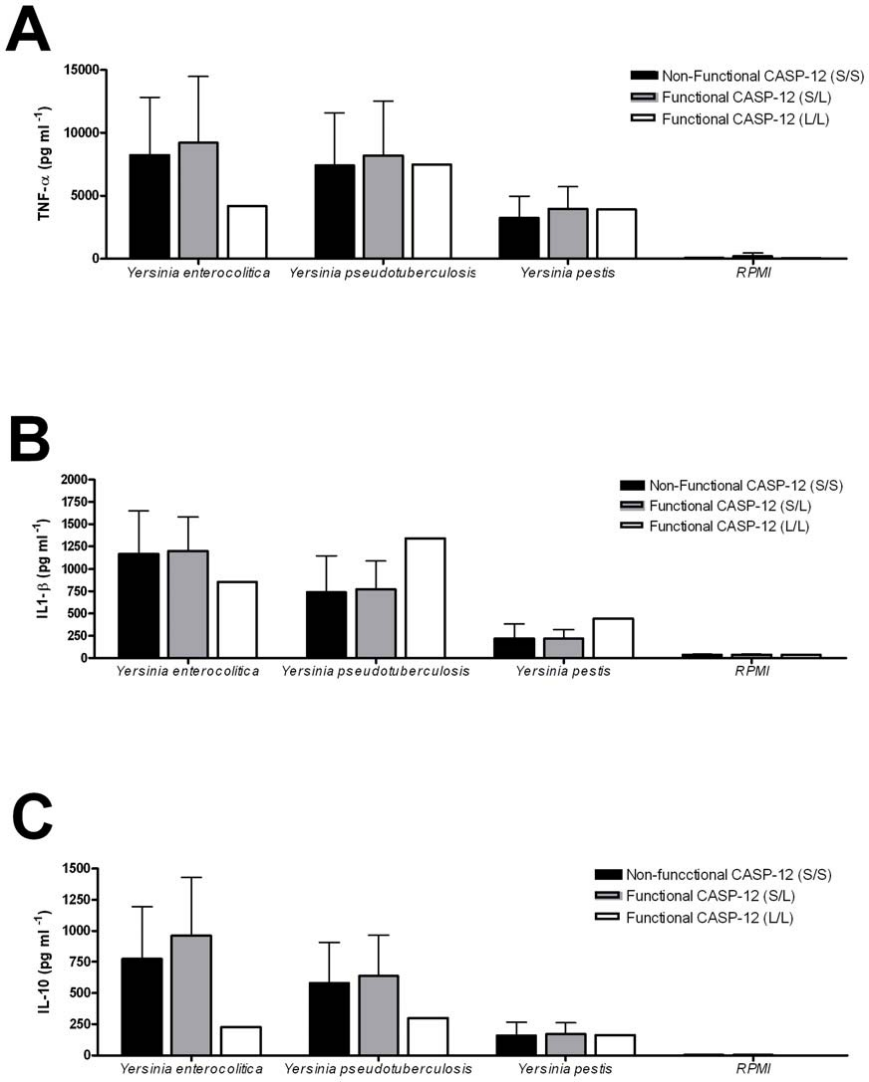
In-vitro cytokine measurements after whole blood stimulation with *Yersinia enterocolitica*, *Yersinia pseudotuberculosis* and *Yersinia pestis*. TNF (A), IL-1β (B) and IL-10 (C) were measured by ELISA after 24-hour stimulation. Volunteers were grouped as homozygous bearing the nonfunctional caspase-12 genotype (S/S), heterozygous bearing the functional caspase-12 and nonfunctional (S/L) and homozygous that bear the functional caspase-12 (L/L). The numbers of volunteers included for each cytokine are S/S = 24, S/L = 22 and L/L = 1. Values represent mean + SD for each group of volunteers. P-values for differences between caspase-12 genotype were calculated with the Mann-Whitney test.

Stimulation with pure TLR4 (LPS) and TLR2 (Pam3Cys) ligands showed no anti-inflammatory role of the functional caspase-12. The lack of inhibition could have been caused by the specific TLR stimuli used in these experiments. Therefore, whole blood from the second volunteer cohort was also stimulated with three pathogenic *Yersinia* species, namely *Y. enterocolitica*, *Y. pseudotuberculosis* and *Y. pestis* (Figure 3). These three *Yersinia* spp were chosen due to their important role as human pathogens. With all three *Yersinia* species no different pro-inflammatory cytokine production was observed between the functional and non-functional caspase-12 individuals (Figure 3). This indicates that the caspase-1 pathway was not affected by the functional caspase-12.

### NOD2/RIP2 pathway

**Figure 4:**
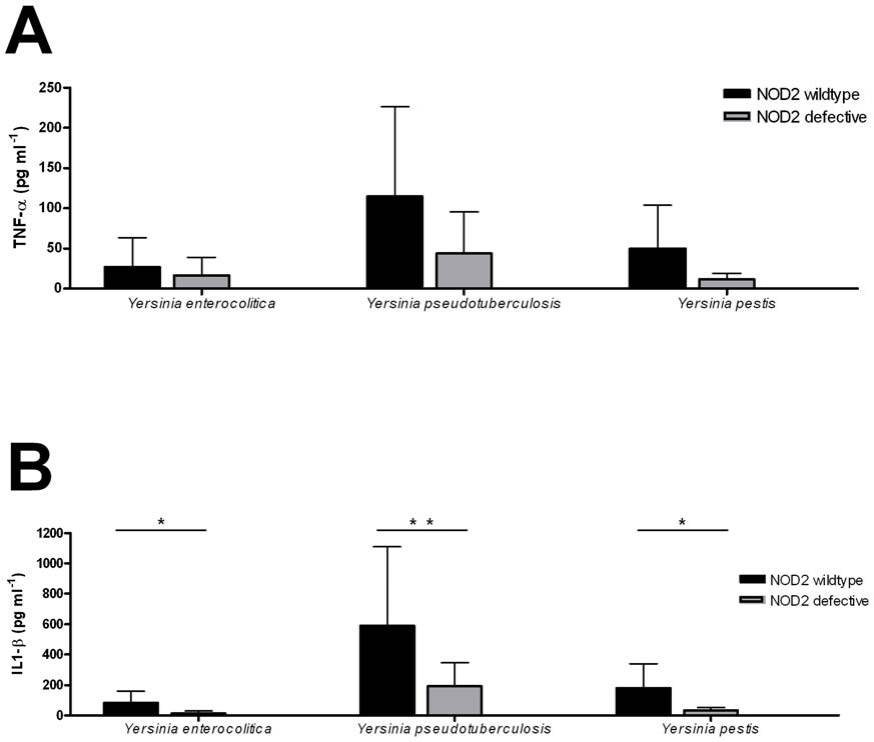
In-vitro cytokine measurements after PBMC stimulation with *Yersinia enterocolitica*, *Yersinia pseudotuberculosis* and *Yersinia pestis*. Caucasian individuals bearing a normal (NOD2 wt, N = 10) or homozygous for the 3020insC NOD2 mutation (NOD2 del, N = 5) were stimulated with the various *Yersinia spp*. TNF (A) and IL-1β (B) were measured by ELISA after 24-hour stimulation. Values represent mean + SD for each group of volunteers. P-values for differences between caspase-12 genotype were calculated with the Mann-Whitney test. *P<0.05, **P≤0.01.

Beside the caspase-1 pathway it has been shown that caspase-12 inhibition of the NOD2/RIP2 pathway is important for the lower pro-inflammatory cytokine release[17]. To test if the NOD2/RIP2 pathway was involved in the innate immune response against *Yersinia* spp. we preformed stimulations with the same three *Yersinia* spp. in cells isolated from individuals homozygous for the 3020insC NOD2 mutation (Figure 4). It was observed that the individuals homozygous for the 3020insC NOD2 mutation display a tendency towards reduced TNF-α production (Figure 4A) and a significant lower IL1-β production after stimulation with all three *Yersinia* spp (Figure 4B). This demonstrates that the NOD2 receptor does play an important role in *Yersinia* recognition by the innate immune system. Secondly, involvement of the NOD2/RIP2 pathway during *Yersinia* spp recognition also indicates that if functional caspase-12 would have inhibited the pro-inflammatory response through the NOD2/RIP2 pathway, a reduction in IL-1β production induced by Yersinia would have been expected, and that was not the case (Figure 3B).

## Discussion

Caspase-12 is a member of the caspase family of cysteine proteases that has been suggested to exert inhibitory effects on proinflammatory cytokines. The only populations that retain a sizeable proportion of individuals with active caspase-12 are those in Sub-Saharan Africa. The aim of the present study was to assess the effect of caspase-12 on the cytokine response of cells isolated from volunteers of African origin, after stimulation with bacterial stimuli. These experiments revealed that functional caspase-12 had no inhibitory effect on the pro-inflammatory response (Figure 1). In a small previous study performed in cells isolated from 18 African Americans (eight homozygous for the non-functional allele, eight heterozygous, two homozygous for the functional caspase-12), Saleh *et al* suggested that a functional caspase-12 decreased cytokine production [11;15].

Because our data from the first field study clearly differed from this previous investigations we repeated our experiments in cells isolated from volunteers from a second independent African population from the same region (Table 1, Figure 2). The results from the second study support the conclusion that caspase-12 had no modulatory effect on the TLR-stimulated production of cytokines.

In the first cohort of volunteers, we did observed an elevated IL-1β production in individuals with an active caspase-12. Interestingly, in a colonic inflammation mouse model Leblanc *et al* also found that mice with a functional caspase-12 had a higher production of pro-inflammatory cytokine on day 14, although no differences in the production of the pro-inflammatory cytokines were observed early in the process [17]. However, this effect on IL-1β production could not be reproduced in the second field study. Therefore, the overall conclusion of these two studies must be that caspase-12 is unlikely playing a major role in the modulation of the inflammatory reaction induced by LPS or bacterial lipopeptides.

Our first set of experiments used specific TLR4 and TLR2 ligands to elicit inflammation. However, bacterial pathogens are much more complex structures, and an effect of caspase-12 during stimulation with intact microorganisms could still be envisaged. In support of this hypothesis, it has been reported that individuals with a functional caspase-12 are more susceptible to sepsis [11]. Therefore, we wanted to assess whether stimulation of cells with various caspase-12 genotypes with complex Gram-negative microorganisms resulted in differences in pro-inflammatory cytokines. We performed stimulations with the Gram-negative bacteria *Y. pestis*, the agent of bubonic plague, an infection that is well-known to have induced an important evolutionary pressure on Europe and Asian populations (Figure 3) [18]. In addition, we also stimulated cells with *Y. enterocolitica*, an important pathogene of the digestive tract, and with *Y. pseudotuberculosis* the recent ancestor of *Y. pestis* [18]. Previous studies have shown the role of TLR2 and TLR4 for *Y. pestis* recognition [19]. In our stimulation of individuals with the homozygote 3020insC NOD2 mutation we demonstrate the involvement of the NOD2 pathway during *Yersinia* spp recognition (Figure 4). The fact that both TLR and NOD2 pathways have been proposed to be modulated by caspase-12, makes *Yersinia* therefore the ideal bacterial model for the assessment of caspase-12 effects.

Important in the recognition of *Yersinia* strains is the recognition through TLR4. Montminy et al. showed that *Y. pestis* actively changes its LPS structure after the transmission to humans [20]. By doing this, *Y. pestis* actively avoids TLR4 recognition and the innate immune response necessary for the clearance. *Yersinia* also manipulates the innate immune system of hosts with a mechanism known as the type III secretion system [21]. Hereby Yop proteins are secreted and interfere with the inflammation response. These proteins take control of host cells by inhibiting the caspase-1-mediated maturation and IL-1β release [22]. This indicates that both NFκB and caspase-1 signaling pathways are involved in the recognition of *Yersinia*. However, our experiments performed in a large number of volunteers bearing a functional caspase-12 do not show any modulation of *Yersinia*-induced cytokines, and thus of the TLR/NOD2 pathways.

Earlier studies on the distribution of caspase-12 genotypes have shown important differences between populations [5;6], but it is unclear how environmental pressure caused selection of certain alleles, and lead to loss of caspase-12 function in the majority of individuals [23]. Our study in two African populations does not support a role for caspase-12 in the modulation of the pro-inflammatory response after TLR challenge. Similarly, no effect of caspase-12 on *Yersinia* stimulation of cytokines was observed. Phylogenetic analyses show that structurally, caspase-12 is grouped with the other inflammatory caspases, but the potential mechanisms through which caspase-12 potentially modulate the inflammation are still not well understood [24;25]. Most data available to date have been obtained in mice defective in caspase-12, that showed a higher cytokine response and increased resistance in bacterial models [15;17]. These data seem to be supported by a small study in humans, suggesting high cytokine responses in humans with inactive caspase-12 [11]. Unfortunately, this study has not been independently confirmed and seem to be contradicted by our data. The cause of the differences between the study of Saleh and colleagues and the present study are unclear. In our study we have performed a dose-response curve of LPS concentrations (Figure 1), and therefore it is unlikely that differences in LPS concentrations could explain the discrepancy. We have also preformed the experiments in a larger numbers of individuals than the original study of Saleh *et al*, and the results were verified in a second cohort. Ethnic differences between the African American volunteers in the study of Saleh *et al* and the Mali population investigated in our study could account for some differences, although the theoretic basis for this is unclear. The only additional study investigating the role of caspase-12 in infections found no correlation between caspase-12 and hepatitis C virus infection [26]. It is possible that caspase-12 may interact also with other PRRs than TLR4 and NOD2. However, by using whole microorganisms as *Yersinia* spp., that very likely stimulates also TLR2, TLR5, TLR9 (etc), we practically exclude an influence of caspase-12 on other important PRR pathways as well.

In summary, the data of the present study show no regulatory role of human caspase-12 on the inflammation induced by TLR ligands or *Yersinia* spp. Although a role of caspase-12 for the susceptibility to sepsis can still be envisaged (which has to be confirmed in larger studies), this is highly unlikely to be mediated through modulation of cytokine response. Alternatively, the selection found in European and Asian populations on the non-functional caspase-12 may have not been caused by sepsis at all, and other biological explanations should be sought.

## Materials and Methods

### Volunteers

Blood samples were collected in the Koro district of Mali, West Africa during two fieldtrips in September 2006 and April 2007. These studies are part of the investigational into interethnic differences in susceptibility to malaria, as described in detail elsewhere [27]. From these volunteers we only included the healthy individuals without any suspicion or diagnosis for malaria. Approval for the study was provided by the institutional review board of the University of Bamako (N°0527/FMPOS).

A total of 10 Dutch Caucasian controls and 5 Dutch Caucasian volunteers homozygous for the loss-of-function 3020insC NOD2 mutation were used for the stimulation experiments investigating the role of NOD2 in the recognition of *Yersinia*. The characteristics of the NOD2-defective individuals are described elsewhere [28].

### Cytokine stimulation assays

Venous whole blood was collected into 10 ml heparin tubes (BD, Plymouth, UK) Blood from healthy individuals was diluted to a final concentration of 1∶5 in RPMI 1640 medium (containing 1% glutamine, 1% pyruvate and 1% gentamicin). Stimulation was performed with control medium, highly purified lipopolysaccharide (*Escherichia colli* 055:B5, sigma, extra purified according to Hirshfeld *et al*. [29]) at various concentrations (1, 10 and 100 ng/ml), and the lipopeptide Pam3Cys (10 µg/ml, EMC Microcollections, Erlaugen). Heat-Killed *Yersinia enterocolitica* (clinical isolated), *Yersinia pseudotuberculosis*, serotype O1 (strain YERS0068) and *Yersinia pestis antiqua*, biovar Antiqua (strain BD94-oo544), were used for the stimulation experiments at a final concentration of 1×10^4^/ml. *Yersinia* strains were cultured in BHI-broth (Tritium, Eindhoven, The Netherlands) for 48 hours at 37°C. The cells were resuspended in milliQ and heat treated at 95°C for 30 minutes. Colony forming units (CFU) and loss of viability of the cells after heat treatment were determined by plating appropriate dilutions on blood agar (Biotrading, Mijndrecht, The Netherlands) followed by incubation at 37°C for 48 hours. After 24 hours of stimulation at 37°C, supernatants were collected and stored at −80°C until cytokines measurements were preformed.

For the stimulations of cells isolated from the NOD2-defective individuals, peripheral blood mononuclear cells (PBMCs) were isolated by density gradient centrifugation on Ficoll, washed three times in cold RPMI, counted and resuspended in complete culture medium. PBMCs (final concentration 2.5×10^6^/ml) were stimulated with three *Yersinia* strains in 96-well round-bottom plates for 24 hours at 37°C. After stimulation, supernatants were collected and directly used for cytokine measurement.

### Cytokine measurements

IL-10 and IL1-β cytokine concentrations were measured by sandwich ELISA (Sanquin, Amsterdam, The Netherlands). TNF-α production was measured by a specific ELISA, as previously described [30].

### Caspase-12 genotypic determination

DNA for all volunteers was extracted from whole blood using the Puregene (Gentra Systems, Minneapolis, MN) isolation kit. Flanking primers of the surrounding T125C polymorphism region and methodology was adopted as described by Saleh *et al* [11]. Sequencing was performed at the sequence faculty at the department of human genetics Nijmegen on the 48-capillary 3730 sequencer (Applied Biosystems). Genotypes of the volunteers were analyzed and determined using the software 4Peaks by A. Griekspoor and T. Groothuis (mekentosj.com).

### Statistical analysis

Data were analysed in SPSS (Rel.16; SPSS, Chicago, IL). Genotypic distribution between 2006 and 2007 was assessed by χ^2^-test. Differences in cytokine response to the stimuli between groups were analysed by Mann-Whitney. P-values<0.05 were considered statistically significant in all analysis.
